# A worldwide cross-sectional survey seeks the current global practice patterns on perioperative management in elective shoulder replacement surgery

**DOI:** 10.1016/j.jseint.2026.101758

**Published:** 2026-07-06

**Authors:** Maciej J.K. Simon, Farhad O. Moola, Jennifer Coghlan, Osvandre Lech, Carlos Henrique Ramos, Njalalle Baraza, Hadrian Platzer, Simone Gantz, Andreas Seekamp, Babak Moradi, Roger van Riet, Simon N. Bell, William D. Regan

**Affiliations:** a Department of Orthopaedics and Trauma Surgery, University Medical Center Schleswig-Holstein, Kiel, Germany; b Department of Orthopaedics, Chan Gunn Pavilion, Allen McGavin Sports Medicine Clinic, University of British Columbia, Vancouver, BC, Canada; c Melbourne Shoulder and Elbow Centre, Sandringham, VIC, Australia; d Department of Orthopaedics, University of British Columbia, Vancouver, BC, Canada; e Fraser Orthopaedic Institute, New Westminster, BC, Canada; f Department of Surgery, School of Clinical Sciences, Monash Health Monash University, Melbourne, VIC, Australia; g Instituto de Ortopedia e Traumatologia, Hospital São Vicente de Paulo, Passo Fundo, RS, Brazil; h Departamento de Ortopedia, Universidade Federal do Paraná, Hospital de Clínicas, Curitiba, PR, Brazil; i Section of Orthopaedics, Aga Khan University Hospital, Nairobi, Kenya; j Orthopedic Research Center, Kiel University, Kiel, Germany; k Department of Orthopaedic Surgery, Antwerp, Belgium; l Department of Orthopaedic Surgery, University Hospital Antwerp, Edegem, Belgium

**Keywords:** Shoulder arthroplasty, Perioperative management, Online questionnaire, Discharge management, Sling use, Pre-operative planning

## Abstract

**Background:**

Perioperative management in elective shoulder arthroplasty varies widely, with no universal consensus on best practices. This study aimed to characterize current global standards of perioperative care, including pre-operative, intraoperative, and post-operative management, among orthopedic surgeons performing elective shoulder replacement surgery, with the aim of contributing to a global standard of care.

**Methods:**

A comprehensive online questionnaire was designed and anonymously distributed to orthopedic societies on each continent. Participants were cross-sectionally surveyed with the goal of identifying current global pre-operative, intraoperative, and post-operative practices in patient care and discharge management.

**Results:**

Eighteen societies from 5 continents participated in the survey. A total of 977 orthopedic surgeons completed the online questionnaire. The data analysis reveals that routine pre-operative advanced imaging studies are not standard with either computed tomography scan (50%) or magnetic resonance imaging scan (39%) preferred. Pre-operative planning software was not routinely used (31%). A chlorhexidine wash is part of the presurgical preparation for 52% of participants, but 23% do no prewash. Beach-chair or lazy beach-chair is the preferred surgical position (99%), and the deltopectoral approach remains the most standard approach (91%). Perioperative antibiotic use is nearly universal (99%), whereas routine tranexamic acid use remains moderate (43%). Post-operative sling use varies from 4-6 weeks including range of motion restrictions. Same-day surgery accounts for only 4% of discharge practices, while 32% discharge on the first post-operative day. Sixty-four percent of respondents discharge patients after several in-hospital nights.

**Conclusion:**

Survey data show marked homogeneity in intraoperative practices (eg, surgical approach, patient positioning, and antibiotic use). However, pre-operative and post-operative survey results, including planning software use, post-operative immobilization, and discharge times, showed substantial variation. These findings highlight the need for further research and consensus to establish evidence-based perioperative protocols in elective shoulder replacement surgery.

Elective shoulder replacement surgery has become an increasingly common and a reliable intervention for patients with degenerative or traumatic shoulder pathology.^46^ Over the past 4 to 5 decades, advancements in prosthesis design have been made. These changes include the development of reverse and anatomic systems, which have significantly improved clinical outcomes.^51^ Innovations in implant materials, such as osteoinductive surfaces, improved tribology including advanced polyethylene liners,^44^ ceramic heads,^4^^,^^24^^,^^43^ and pyrocarbon hemi-prostheses, have reduced wear and enhanced fixation.^20^ Moreover, the trend toward shorter ± stemless prosthesis designs reflects ongoing efforts to minimize risk while optimizing surgical outcomes.^43^

While intraoperative steps have remained relatively consistent, surgical technique continues to improve. Retractor design, for example, has evolved to address the unique anatomical challenges of shoulder arthroplasty, with many instruments now tailored specifically for this indication.^53^ The adoption of advanced pre-operative imaging modalities, 3-dimensional planning software, and navigation tools has grown, enhancing the accuracy of component placement.^6^^,^^39^ There is research interest in standardizing pre-operative and post-operative care pathways, including pain management, immobilization strategies, and discharge planning.^2^^,^^17^^,^^32^

However, to date, there is a discrepancy regarding many perioperative factors, for example, discharge management, the length and type of post-operative sling use, and other factors sought after in this survey. We aimed to survey current practice preferences among orthopedic societies worldwide to identify areas of consensus and divergence, with the ultimate goal of establishing current evidence based on global practice patterns, providing a basis for further international consensus studies for perioperative management of elective shoulder replacement.

## Materials and methods

### Questionnaire

We developed a comprehensive, anonymous, cross-sectional online survey using the Qualtrics platform (Qualtrics, Provo, UT, USA) focused on perioperative management in elective shoulder arthroplasty. Institutional review board approval was obtained (D518/22). All participants provided informed consent. The study was carried out in accordance with the latest Declaration of Helsinki.

No IP addresses were tracked. Additionally, access to the platform was limited to the first author. Multiple participations were automatically blocked via survey platform settings. Time to complete the questionnaire was approximately 15 minutes.

The survey was developed by some of the current authors who are from 3 different continents and have combined well over a century of experience in this field of surgery. The questionnaire was in a multistep process that was thoroughly streamlined. Thereafter, each survey developer conducted pretesting with a small group of colleagues. Subsequently, further adjustments and fine-tuning were performed.

The observational survey comprised more than 40 questions, capturing surgeon demographics, pre-operative evaluation (including rotator cuff assessment, imaging, and planning software usage), intraoperative techniques (patient positioning, surgical approach, antibiotic and tranexamic acid [TXA] use, subscapularis management, closure principles), and post-operative care (discharge process, immobilization orthoses, radiographic follow-up, and rehabilitation protocols) (Supplementary data). Questions were dependent on type: mostly single-answer, but also multiple-answer. There were a few specific questions distinguishing between prosthesis types. One of the following is concerned with subscapularis management, another with post-operative sling use, and the last one with whether different rehabilitative protocols are used for different shoulder replacement types. All other questions apply to both types of shoulder replacements.

### Study cohort

Eighteen national and international orthopedic or shoulder and elbow societies from 5 continents were invited to participate. Following society approval, the survey was disseminated by society secretaries to their active members, with 2 reminder e-mails sent at 3-week intervals. The survey was open from early 2022 until the beginning of 2024.

**Figure 1:**
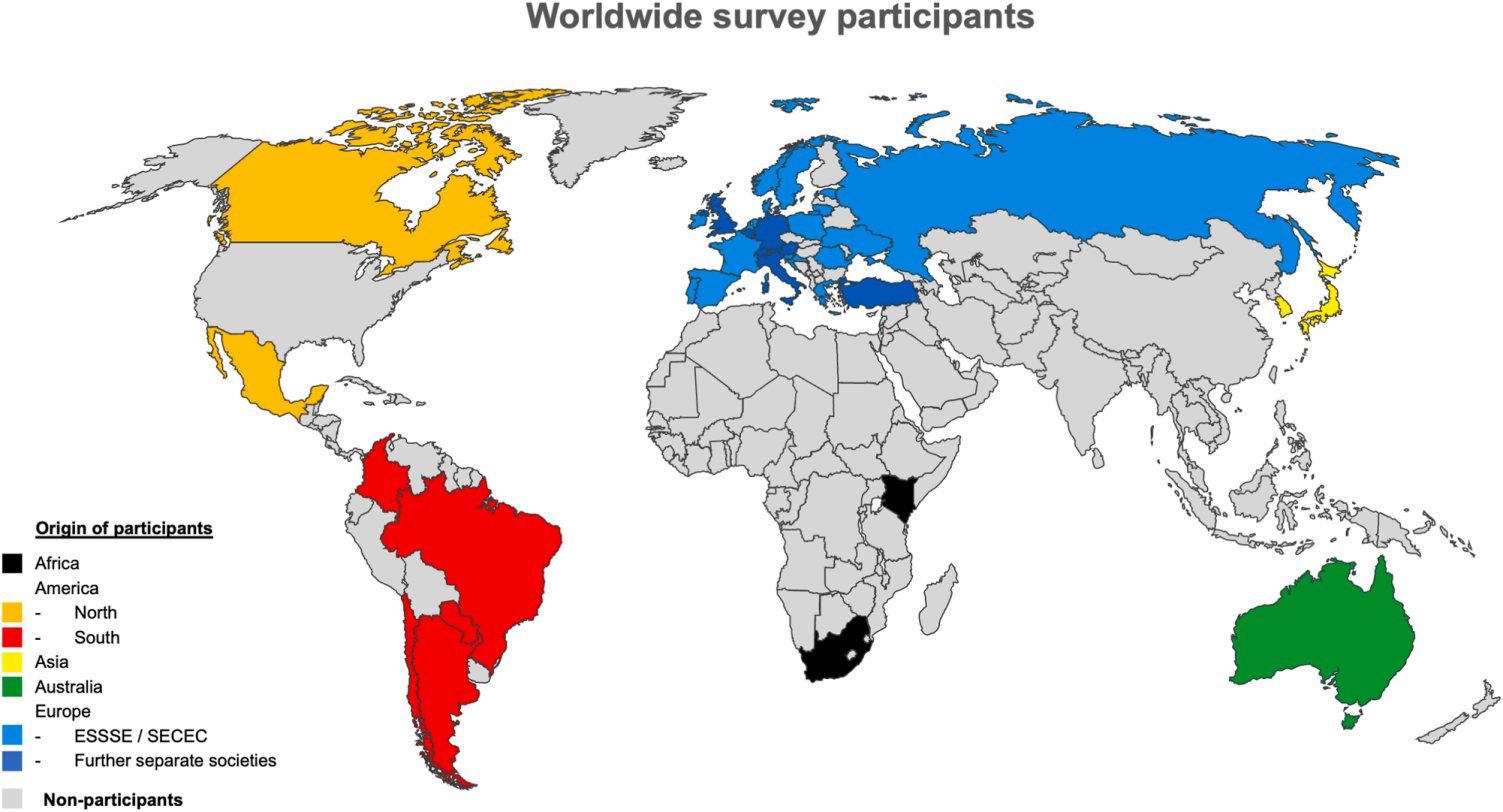
World map demonstrating the distribution of survey participants highlighting the wide reach of the survey.

The following societies participated in the survey (Fig. 1):

- Argentine: Argentine Society of Shoulder and Elbow Surgery (ASSES)
- Australia: Shoulder and Elbow Society of Australia (SESA)
- Belgium: Belgium Elbow and Shoulder Society (BELSS)
- Brazil: Brazilian Society of Shoulder and Elbow Surgery (Sociedade Brasileira de Cirurgia do Ombro e Cotovelo – SBCOC)
- British: British Shoulder & Elbow Society (BESS)
- Canada: Canadian Shoulder and Elbow Society (CSES)
- Chile: Sociedad Chilena de Ortopedia y Traumatología ‑ Comité de Hombre y Codo (SCHOT)
- Colombia: Sociedad Colombiana de Hombre y Codo (SCHOC)
- DACH: German-speaking shoulder and elbow society – D-A-CH Vereinigung für Schulter und Ellenbogenchirurgie (DVSE)
- Europe: Société Européenne de Chirurgie de l’Epaule et du Coude (SECEC)/European Society for Surgery of the Shoulder and Elbow (ESSSE)
- Italy: Italian Shoulder and Elbow society (Società italiana di chirurgia della spalla e del gomito ‑ SICSEG)
- Japan: Japan Shoulder Society (JSS)
- Kenya: Kenya Orthopaedic Association (KOA)
- Korea: Korean Shoulder and Elbow Society (KSES)
- Mexico: Sociedad Mexicana de Cirujanos de Hombre y Codo (SMCHC)
- Paraguay: Sociedad Paraguaya de Hombre y Codo (SPHOC)
- South Africa: South African Shoulder and Elbow Society (SASES)
- Türkiye: Turkish Society of Shoulder and Elbow Surgery/Türkiye omuz ve dirsek cerrahisi dernegi (TODCD)

### Data

A total of 7,506 active society members were sent an invitation participation link to the survey. The responses were anonymously tabulated, depicted in graphs, or reported in aggregate form. Categorical variables were reported in sample size and/or percentiles. Only fully completed questionnaires were included in the analysis.

### Statistical analysis

Data were analyzed using SPSS Statistics (Version 31.0.0.0 (117) IBM Corp., Armonk, NY, USA). Descriptive statistics were calculated as frequencies and percentages. Pearson's chi-square tests were used to assess associations between categorical variables, and Cramér's V was reported as a measure of effect size where appropriate. For ordinal variables, Spearman's rank correlation coefficient was used. Differences between regions or income-level groups were assessed using chi-square tests. Additional exploratory subgroup and cross-variable analyses were performed to further examine associations between perioperative practice patterns and provider-level or system-level characteristics. Missing data were handled by pairwise deletion. All tests were 2-tailed, and a level of *P* < .05 was considered statistically significant.

## Results

A total of 977 completed responses (excluding 222 incomplete surveys) were received from 7,506 invited members, representing an average response rate of 16.95% (Fig. 1). The majority of respondents were male (n = 903; 92.43%) and aged 40-60 years (n = 614; 62.85%) (Table I). Most were fellowship-trained in shoulder surgery (n = 789; 80.76%) but the majority performed less than 50 elective shoulder arthroplasties annually (n = 788; 80.66%).

**Table I:** Participants' societies, ages, genders, work settings and experiences, and arthoplasty numbers per year

Ages																			
	Argentine	Australia	Belgium	Brazil	British	Canada	Chile	Colombia	DACH	Europe	Italy	Japan	Kenya	Korea	Mexico	Paraguay	South Africa	Türkiye	Total
<30 yrs	0	0	0	3	0	0	0	0	0	2	1	0	0	0	0	0	0	0	6
30-40 yrs	4	1	11	54	8	6	3	2	28	29	14	18	3	5	17	0	3	5	211
>40-50 yrs	5	10	9	65	45	10	2	0	49	42	10	60	5	6	5	1	11	2	337
>50-60 yrs	2	16	5	24	35	13	1	3	56	48	15	33	2	7	3	0	11	3	277
>60-70 yrs	2	13	1	22	14	3	0	3	26	22	2	19	0	1	2	0	7	1	138
>70 yrs	0	0	0	2	0	0	0	0	2	2	1	0	0	0	0	0	1	0	8
Total	13	40	26	170	102	32	6	8	161	145	43	130	10	19	27	1	33	11	977

Gender																			
	Argentine	Australia	Belgium	Brazil	British	Canada	Chile	Colombia	DACH	Europe	Italy	Japan	Kenya	Korea	Mexico	Paraguay	South Africa	Türkiye	Total
Male	12	37	20	163	92	28	6	8	151	124	38	127	9	19	25	1	32	11	903
Female	1	3	6	7	9	4	0	0	10	21	4	3	1	0	2	0	1	0	72
Other	0	0	0	0	0	0	0	0	0	0	1	0	0	0	0	0	0	0	1
Prefer not to answer	0	0	0	0	1	0	0	0	0	0	0	0	0	0	0	0	0	0	1
Total	13	40	26	170	102	32	6	8	161	145	43	130	10	19	27	1	33	11	977

Work setting (multiple answers possible)																			
	Argentine	Australia	Belgium	Brazil	British	Canada	Chile	Colombia	DACH	Europe	Italy	Japan	Kenya	Korea	Mexico	Paraguay	South Africa	Türkiye	Total
University hospital	4	13	3	62	46	21	3	4	23	62	12	41	8	11	7	1	5	7	333
General hospital	7	13	18	85	45	13	3	0	77	52	27	60	1	5	5	0	1	1	413
Private hospital	6	32	8	156	42	1	5	4	55	52	10	33	2	3	19	0	26	6	460
Ambulatory surgery center	2	2	0	33	3	6	0	3	25	3	1	0	0	0	1	0	0	0	79
Private practice	7	31	2	130	20	3	0	4	33	26	2	5	1	0	17	0	12	0	293
Other	1	0	0	9	1	1	1	0	4	1	0	1	1	0	0	0	0	0	20
Total	27	91	31	475	157	45	12	15	217	196	52	140	13	19	49	1	44	14	1,598

Work experience in yrs																			
	Argentine	Australia	Belgium	Brazil	British	Canada	Chile	Colombia	DACH	Europe	Italy	Japan	Kenya	Korea	Mexico	Paraguay	South Africa	Türkiye	Total
<5 yrs	0	0	6	26	3	5	2	0	2	15	7	2	1	3	5	0	2	2	81
>5-10 yrs	4	1	5	32	6	5	1	2	15	19	9	11	3	5	12	1	2	3	136
>10-20 yrs	6	9	7	55	37	12	2	1	53	42	12	44	3	6	5	0	10	2	306
>20-30 yrs	2	21	8	39	35	7	1	2	57	49	12	47	3	5	3	0	11	3	305
>30 yrs	1	9	0	18	21	3	0	3	34	20	3	26	0	0	2	0	8	1	149
Total	13	40	26	170	102	32	6	8	161	145	43	130	10	19	27	1	33	11	977

Fellowship experience																			
	Argentine	Australia	Belgium	Brazil	British	Canada	Chile	Colombia	DACH	Europe	Italy	Japan	Kenya	Korea	Mexico	Paraguay	South Africa	Türkiye	Total
Yes	13	35	24	161	95	32	5	6	94	125	35	90	6	16	24	1	18	9	789
No	0	5	2	9	7	0	1	2	67	20	8	40	4	3	3	0	15	2	188
Total	13	40	26	170	102	32	6	8	161	145	43	130	10	19	27	1	33	11	977

Arthroplasties per yr																			
	Argentine	Australia	Belgium	Brazil	British	Canada	Chile	Colombia	DACH	Europe	Italy	Japan	Kenya	Korea	Mexico	Paraguay	South Africa	Türkiye	Total
<20	11	3	3	133	27	3	2	3	34	33	18	87	9	7	19	1	8	6	407
>20-50	1	26	12	36	65	12	4	5	72	58	17	34	1	10	6	0	19	3	381
>50-100	1	8	7	1	9	11	0	0	40	38	5	6	0	2	2	0	5	2	137
>100-200	0	3	4	0	1	6	0	0	13	14	3	3	0	0	0	0	1	0	48
>200-300	0	0	0	0	0	0	0	0	1	2	0	0	0	0	0	0	0	0	3
>300	0	0	0	0	0	0	0	0	1	0	0	0	0	0	0	0	0	0	1
Total	13	40	26	170	102	32	6	8	161	145	43	130	10	19	27	1	33	11	977

*DACH*, German-speaking shoulder and elbow society.

### Pre-operative imaging and planning

**Figure 2:**
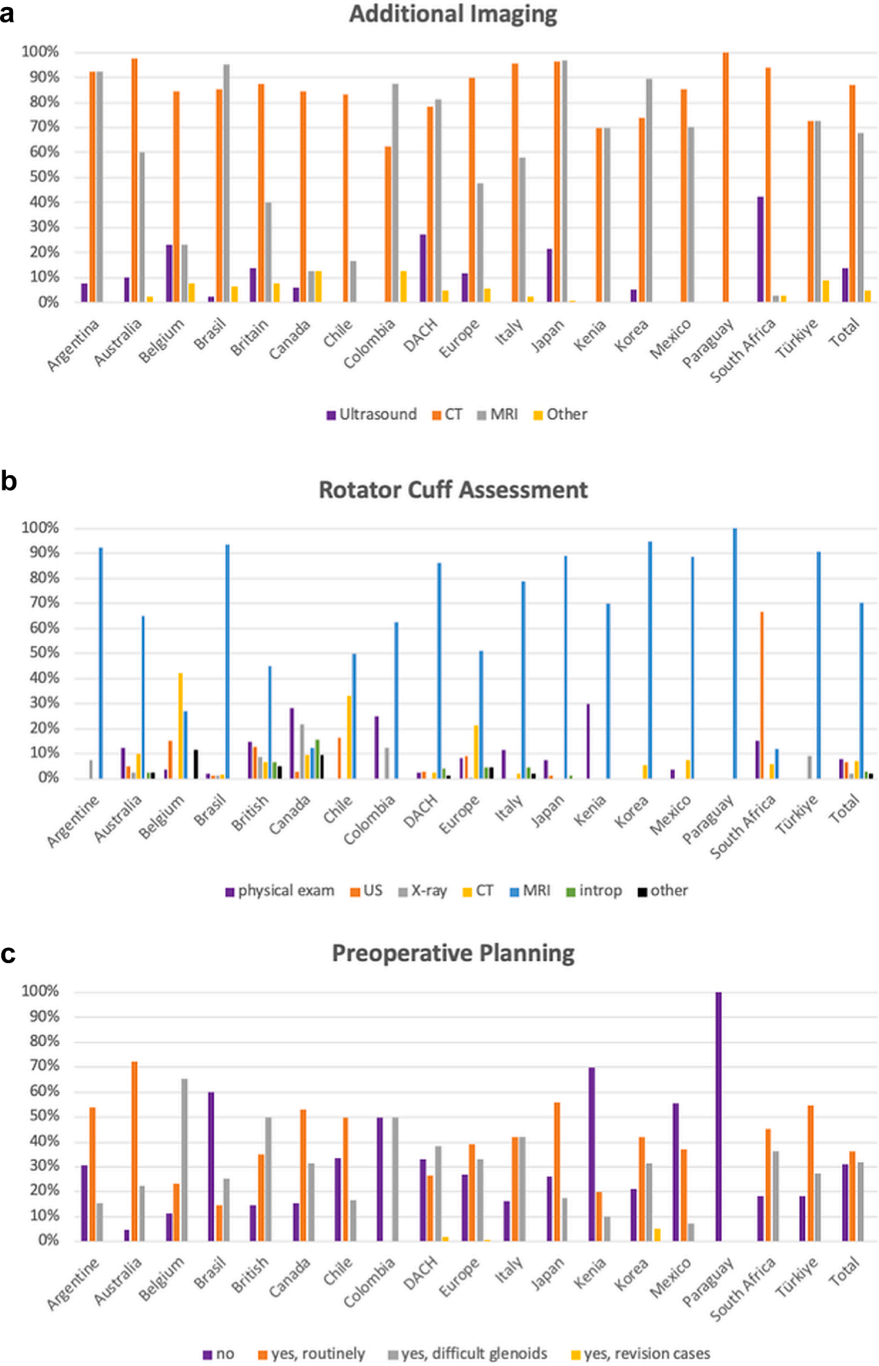
Part **a**: Additional imaging demonstrating an overall favor for CT (50%) vs. MRI (39%) scans (multiple-choice responses). Part **b** demonstrates rotator cuff assessment preferences among surgeons in favor for MRI use (single-choice question). Part **c**: Routine pre-operative planning software use is overall indecisive with just a minor majority for routine planning (single-choice question). *CT*, computed tomography; *MRI*, magnetic resonance imaging.

Standard radiographs were universally obtained, with advanced imaging favoring computed tomography (CT) (n = 850; 50%) over magnetic resonance imaging (MRI) (n = 660; 39%) in this multiple-answer response (Fig. 2*A*). Rotator cuff assessment was predominantly performed with MRI (n = 689; 71%), with only 8% (n = 76) relied solely on physical examination (Fig. 2*B*). Use of pre-operative planning software was heterogeneous: 31% (n = 305) reported no use, 32% (n = 312) reserved it for complex glenoid cases, and 36% (n = 355) used it routinely (Fig. 2*C*). Additional analysis examined the association between planning software use and annual arthroplasty volume. This showed a significant association (Pearson's chi-square = 1,175.301, *P* < .001), with a large effect size (Cramer's V = 0.633). There was also a significant linear-by-linear association (*P* < .001), indicating that the use of planning software increased with higher surgical-volume categories.

### Pre-operative preparation

A pre-operative chlorhexidine skin wash was performed by 52% (n = 511) of surgeons, while 23% omitted any pre-operative skin cleansing. The use of Ioban drapes was common at 56% (n = 678). Only 12% (n = 224) operated with a helmet (multiple-answer response).

Perioperative antibiotic prophylaxis was near universal (n = 970; 99%) in the form of a single shot, up to 24 or 48 hours or even longer, most commonly cefazolin (n = 758; 78%). TXA was used routinely by 43% (n = 416) to manage bleeding, occasionally by 24% (n = 235), and not at all by 33% (n = 319).

### Intraoperative technique

Patient positioning was almost exclusively beach-chair (n = 592; 61%) or lazy beach-chair (n = 376; 38%). The use of an arm holding device varied globally, with the pneumatic arm holder (n = 304; 31%) slightly more popular than none (n = 194; 20%). The deltopectoral approach was preferred by 91% (n = 890) of surgeons, with the cephalic vein typically retracted and protected (n = 920; 94%). The majority managed the biceps with tenodesis (n = 754; 77%), and common soft-tissue releases included the pectoralis major (n = 614; 47%) and the coracoacromial ligament (n = 448; 34%).

Subscapularis management was split between tenotomy (n = 417; 43%) and peel-off (n = 375; 38%), with regional preferences observed: Asian, Australian, and Turkish surgeons favored peel-off, while European surgeons preferred tenotomy. The top 5 globally used retractors are Glenoid, Hohmann, Fukuda, Langenbeck, and Gelpi retractors (multiple-answer response).

The majority of surgeons place the glenoid pin with the use of a standard guide without prior planning (n = 417; 43%). Standard guide with prior planning (n = 224; 23%) is the second most common usage followed by freehanded (n = 145; 15%), freehanding with planning (n = 80; 8%), patient specific instruments (n = 60; 6%), and real time (n = 30; 3%).

### Closure and post-operative care

For anatomic arthroplasty, subscapularis repair was universally performed, most often with transosseous non-absorbable sutures (n = 638; 65%). In reverse arthroplasty, 15% (n = 147) omitted subscapularis repair, while 58% (n = 569) used transosseous non-absorbable sutures for the repair. Local antibiotics were rarely used during closure (n = 118; 12%). Skin closure was most commonly achieved with subcutaneous absorbable sutures (n = 539; 37.9%), often combined with steristrips (n = 226; 15.9%) (multiple-answer response).

Post-operative immobilization use was done with regular (n = 642; 66% vs. n = 623; 64%) and abduction (n = 296; 30% vs. n = 300; 31%) slings in anatomic and reverse replacements, respectively, with the vast majority (n = 800; 82% vs. n = 706; 73%) recommending sling use for a minimum of 3-6 weeks. External rotation and active elevation restrictions were commonly instituted (multiple-answer response). Pain management typically involved non-steroidal anti-inflammatory drugs, opioids for 2-3 days, local cooling devices, and interscalene nerve blocks (Table II) (multiple-answer response).

**Table II:** Post-operative pain management, discharge management, and reason for delay discharge

Post-operative pain management																			
	Argentine	Australia	Belgium	Brazil	British	Canada	Chile	Colombia	DACH	Europe	Italy	Japan	Kenya	Korea	Mexico	Paraguay	South Africa	Türkiye	Total
Interscalene nerve block	21%	24%	33%	13%	35%	25%	18%	37%	26%	27%	24%	34%	20%	17%	19%	33%	21%	16%	25%
Local tnfiltration anesthetic	3%	11%	0%	2%	3%	8%	0%	0%	5%	4%	1%	4%	10%	6%	3%	33%	16%	0%	5%
Opioids for 2-3 d	21%	17%	21%	14%	19%	17%	35%	5%	8%	15%	17%	4%	35%	19%	13%	0%	21%	37%	14%
Opioids for as long as needed	7%	13%	9%	25%	9%	15%	0%	21%	16%	8%	12%	5%	5%	12%	3%	0%	8%	5%	13%
NSAIDs and other non-opioids	34%	20%	18%	22%	26%	19%	35%	26%	29%	33%	21%	42%	30%	27%	37%	0%	25%	32%	28%
Local cooling device	10%	11%	18%	24%	6%	14%	12%	11%	15%	12%	22%	10%	0%	19%	24%	33%	8%	11%	15%
Other	3%	5%	1%	1%	1%	3%	0%	0%	1%	2%	1%	0%	0%	0%	1%	0%	1%	0%	1%

Same-day surgery ‑ discharge management																			
	Argentine	Australia	Belgium	Brazil	British	Canada	Chile	Colombia	DACH	Europe	Italy	Japan	Kenya	Korea	Mexico	Paraguay	South Africa	Türkiye	Total
Yes	0%	0%	0%	1%	6%	44%	0%	25%	0%	3%	0%	5%	0%	0%	4%	0%	0%	0%	4%
No, discharge next morning	85%	18%	27%	52%	75%	53%	67%	63%	0%	42%	5%	1%	40%	5%	41%	0%	42%	0%	32%
No, discharge after 2 nights in hospital	15%	63%	65%	45%	14%	0%	33%	13%	5%	34%	70%	2%	30%	21%	48%	0%	48%	82%	28%
No, after 3-5 nights	0%	18%	8%	2%	2%	0%	0%	0%	72%	13%	26%	2%	30%	16%	0%	0%	9%	18%	18%
No, more than 5 nights (5-10 d)	0%	3%	0%	0%	0%	0%	0%	0%	22%	6%	0%	22%	0%	37%	0%	100%	0%	0%	8%
No, when wound has healed (>10 d)	0%	0%	0%	0%	0%	0%	0%	0%	1%	1%	0%	57%	0%	21%	4%	0%	0%	0%	8%
Other	0%	0%	0%	0%	4%	3%	0%	0%	0%	1%	0%	11%	0%	0%	4%	0%	0%	0%	2%

Reasons for delay discharge																			
	Argentine	Australia	Belgium	Brazil	British	Canada	Chile	Colombia	DACH	Europe	Italy	Japan	Kenya	Korea	Mexico	Paraguay	South Africa	Türkiye	Total
Hospital resources	11%	6%	14%	19%	15%	17%	40%	9%	8%	10%	7%	14%	7%	17%	8%	0%	4%	9%	12%
Culture	21%	25%	20%	13%	23%	21%	0%	27%	11%	19%	20%	32%	29%	20%	28%	0%	15%	27%	19%
Billing system (lower reimbursement)	0%	1%	9%	2%	1%	0%	20%	0%	21%	10%	19%	5%	0%	7%	0%	0%	0%	0%	9%
Anesthesia support	26%	11%	14%	25%	7%	10%	10%	18%	15%	16%	19%	12%	0%	13%	14%	0%	13%	23%	15%
Physio support	0%	13%	18%	6%	16%	13%	10%	9%	22%	14%	13%	19%	21%	10%	14%	100%	19%	23%	15%
Patient travel distances	21%	10%	2%	8%	12%	19%	20%	9%	10%	13%	8%	13%	14%	10%	6%	0%	18%	9%	11%
Other logistics	11%	8%	7%	11%	13%	13%	0%	27%	5%	9%	7%	4%	14%	13%	17%	0%	10%	9%	9%
Other	11%	25%	16%	16%	13%	8%	0%	0%	9%	9%	7%	1%	14%	10%	14%	0%	19%	0%	10%

*DACH*, German-speaking shoulder and elbow society; *NSAIDs*, non-steroidal anti-inflammatory drugs.

### Discharge and follow-up

Discharge practices varied widely. While 4% (n = 36) of surgeons (notably in Canada, Japan, and the United Kingdom) discharged patients on the day of surgery, the majority discharged after one (n = 309; 32%) or 2 nights (n = 273; 28%). Barriers to earlier discharge included customary expectations (n = 366; 19%), anesthesia or physiotherapy support (n = 291 and 298; 15% each), and reimbursement concerns (n = 166; 9%) (multiple-answer response) (Table II). Further analyses demonstrated a significant positive Spearman correlation between discharge category and national GDP/capita (Spearman's rho = 0.326, *P* < .001),^54^ implying that discharge patterns differed systematically according to country income level.

Physiotherapy was the primary means of post-operative rehabilitation (n = 865; 71%) (multiple-answer response). Participants use the same post-operative protocol regardless of prosthesis type 47% (n = 456) of the time while 53% (n = 521) preferring different protocols.

Post-operative radiographs were obtained immediately post-operatively (n = 395; 40%) or the next day prior discharge (n = 452; 46%), with fewer surgeons obtaining further imaging at 6 weeks (n = 605; 26%) and 1 year (n = 498; 22%) post-operatively. Long-term follow-up was common, with 43% (n = 232) of surgeons monitoring patients yearly or if spontaneously needed (n = 854; 87%) (multiple-answer response).

### Additional cross-analyses

Additional analysis of fellowship training and TXA use showed a significant association with a moderate effect size (χ^2^ = 264.130, *P* < .001, Cramér's V = 0.520). This identifies that TXA use patterns differed substantially between respondents with and without fellowship training. Furthermore, fellowship training was significantly associated with several imaging-related variables, but most significantly CT use (χ^2^ = 258.035, *P* < .001).

Societies were categorized into 4 income levels based on the previous year's Gross National Income per capita, using the World Bank Group's yearly World Economic Classification.^54^ Income levels are significantly associated with the following variables. Fellowship training was associated with income level (χ^2^ = 22.962, *P* < .001; Cramér's V = 0.153). Surgical volume showed a significant positive correlation with income level (Spearman's rho = 0.420, *P* < .001). Planning software use was also significantly associated with income level (χ^2^ = 88.863, *P* < .001). Furthermore, discharge timing was significantly associated with income level (Spearman's rho = 0.326, *P* < .001).

## Discussion

The findings from this global survey offer an up-to-date global perspective regarding perioperative management in elective shoulder replacement surgery, reflecting practices across 18 international shoulder societies worldwide. While there is strong consensus on many intraoperative processes, significant variation remains in pre-operative and post-operative processes. These results highlight areas of convergence and divergence and can help advance the field toward further consensus research.

Rotator cuff assessment is overwhelmingly performed with MRI (71%), while CT is slightly more favored than MRI for additional pre-operative glenohumeral joint evaluation (50% vs. 39%). This preference can be due to the surgeon's need to assess bony morphology over the soft tissues, particularly in reverse shoulder arthroplasty, but may also be influenced by the broader global availability of CT scanners compared to MRI scanners, as described by Martella et al.^33^ Their research across 16 Organisation for Economic Co-operation and Development countries demonstrated a steady increase in CT scanner numbers over 2 decades, more prevalent than MRI. Moreover, disparities in imaging resolution persist even among high-income countries.^21^ Similar challenges exist in low- and middle-income countries, where resource limitations, maintenance costs, and shortages of trained personnel may hinder access to high-quality imaging.^18^ The present study surveyed 10 high-income, 7 upper-middle income, and one lower-middle income countries.^54^ This can lead to response-rate bias and is a known methodological limitation in cross-country and cross-cultural online surveys.^16^^,^^50^ Even when advanced imaging is available, overutilization remains problematic, potentially causing delays for patients with urgent needs.^45^^,^^49^

The adoption of 3-dimensional pre-operative planning software remains limited, primarily due to its dependency on high-quality CT imaging plus the initial time investment required, but this may ultimately enhance surgical preparedness.^39^ This is reflected in glenoid pin placement practices, with most surgeons (43%) relying on standard guides or freehand (15%) techniques rather than pre-operative planning.

There is notable unity in intraoperative practices involving minimal capital outlay such as patient positioning (classic or lazy beach-chair), surgical approach (deltopectoral), soft-tissue releases, preferred retractors, and perioperative antibiotic use (99%). The near-universal adoption of perioperative antibiotics can be attributed to the widespread implementation of the World Health Organization Surgical Safety Checklist.^19^^,^^27^^,^^37^^,^^48^ TXA has gained popularity in reducing intraoperative blood loss during shoulder arthroplasty,^28^^,^^38^ although its use is not yet as common as in trauma or hip and knee arthroplasty settings.^9^^,^^22^^,^^26^^,^^35^^,^^41^^,^^42^

Subscapularis management continues to vary worldwide, with tenotomy vs. peel-off techniques being most common. External evidence demonstrates that both techniques yield comparable internal-rotation strength.^30^ Lesser tubercle osteotomy, while more biomechanically stable,^14^ has not demonstrated significant clinical superiority over the other techniques.^3^ Subscapularis-sparing approaches remain less widely adopted, with limited experience reported.^29^^,^^47^

Post-operative immobilization, particularly sling type and duration, also varies considerably among societies. Previous research on abduction (neutral rotation) slings has shown improved outcomes in external rotation and adduction and reduced pain.^8^ However, the present study demonstrates that regular slings remain more common in Europe, while abduction slings are more frequently used in German-speaking countries, plus Japan and Korea. Notably, increased surgeon experience is associated with shorter sling duration, as seen by Freehill et al.^17^ Post-operative pain management in this survey involves the use of various methods. Optimization of this important puzzle piece can improve patient experience and discharge management, particularly with a multimodal opioid-sparing approach.^13^^,^^25^^,^^31^

Discharge practices after shoulder arthroplasty present some of the greatest international variation, as evidenced by survey results. Some surgeons, particularly in Canada, Japan, and the United Kingdom, discharge patients on the day of surgery. The remainder prefers stays of one or 2 nights. Cultural expectations, anesthesia protocols, physiotherapy support, travel distances, hospital resources, and reimbursement systems all influence discharge timing. External evidence suggests that same-day discharge is both feasible and safe with appropriate patient selection,^11^^,^^23^^,^^40^^,^^52^ but societal expectations and infrastructural limitations often preclude this practice. Outpatient care resources, including physiotherapists, nurses, and social workers, are critical, and their absence, particularly in high-income countries facing workforce shortages, can delay discharge.^1^^,^^2^^,^^10^^,^^36^ Moreover, financial incentives embedded in national reimbursement systems may discourage early discharge, as observed in German-speaking countries. A non-negligible factor emerging from these results is the society's country income level, commonly classified by the World Bank according to gross national income per capita.^7^ The present data demonstrate that high-income settings are significantly associated with fellowship training, higher surgical volume, advanced imaging, use of planning software, TXA use, and structured discharge management. This is highly relevant, as global surgical literature consistently shows that access to safe, specialized, and technologically supported surgery is strongly shaped by national resource availability.^5^^,^^34^ Thus, income level should not be interpreted as a minor demographic descriptor, but as a structural determinant of perioperative management in elective shoulder arthroplasty. While this may limit direct generalizability, it strengthens the survey by exposing real-world disparities that must be acknowledged rather than ignored. Future studies should therefore weight or stratify outcomes by income level to distinguish true international consensus from resource-driven practice patterns. This is particularly important for technologies such as 3-dimensional planning and navigation, which are increasingly relevant in shoulder arthroplasty, and for perioperative adjuncts such as TXA, whose benefits in reducing blood loss have been supported in shoulder arthroplasty literature.^12^^,^^38^ Only by accounting for income level can future global standards become both scientifically valid and practically applicable across different healthcare systems.^50^

This study has several limitations that warrant consideration. First, participation was voluntary and response rates were moderate, introducing the possibility of non-response bias. Surgeons with a particular interest in shoulder arthroplasty, perioperative pathways, or academic engagement may have been more likely to participate, potentially influencing the observed distribution of practices. In the future, the non-response bias needs to be minimized with shorter or split, more targeted, and less time-consuming surveys, potentially with a minimum number of shoulder arthroplasty cases per surgeon per year to obtain a higher response quality.

Second, participation was not universal, and some major societies were not represented (eg, American Shoulder and Elbow Surgeons). In addition, differences in society size and regional weighting were not adjusted for, which may limit the representativeness of the sample. In particular, lower-resource settings and non-academic environments may be under-represented, restricting the generalizability of the findings across all global practice contexts.

Third, the survey instrument did not undergo formal psychometric or conceptual validation. Although the questions were designed to be clinically intuitive and directly applicable, variations in interpretation across different healthcare systems, languages, and cultural contexts cannot be excluded.^16^^,^^50^

Fourth, the analyses are exploratory and based on cross-sectional data. While statistically significant associations were identified using chi-square tests, effect size measures, and rank-based correlations, no multivariable adjustment for potential confounding factors was performed. As a result, the findings do not allow causal inference, and observed associations may reflect underlying structural factors such as healthcare system organization, resource availability, or case mix rather than independent effects of individual variables.

Fifth, some subgroup analyses included categories with low cell counts, which may affect the robustness of individual estimates despite the overall large sample size. These results should therefore be interpreted with appropriate caution.

Finally, the extended survey period may introduce temporal bias, particularly for rapidly evolving practices such as the adoption of pre-operative planning software or TXA. As clinical practice continues to evolve, the reported patterns should be understood as reflecting a dynamic rather than static landscape.

Despite these limitations, this study provides a comprehensive international overview of current perioperative practices in elective shoulder arthroplasty (Table III). By capturing both areas of broad consensus and substantial variability, it offers a valuable, hypothesis-generating foundation for future comparative studies, consensus processes, and guideline development across diverse healthcare systems.

**Table III:** Survey's top answers

Items	Assessment	Common thread	Percentages	Question type
1	Biometric data assessment			Single-choice response
2	Additional imaging	CT vs. MRI	50% vs. 39%	Multiple-choice response
3	Rotator cuff assessment	MRI	71%	Single-choice response
4	Pre-operative planning	Yes, routinely	36%	Single-choice response
5	Patient positioning	Beach chair	61%	Single-choice response
6	Armholder	Pneumatic armholder	31%	Single-choice response
7	Pre-operative skin preparation	Chlorhexidine	52%	Single-choice response
8	Additional pre-operative preparation	Ioban drapes	56%	Multiple-choice response
9	Antibiotics - when?	Up to 24 h post-operatively	42%	Single-choice response
10	Antibiotics - what?	Cefazolin	78%	Single-choice response
11	Tranexamic acid (TXA)	Yes, routinely	43%	Single-choice response
12	TXA - when?	Start	72%	Single-choice response
13	TXA - how?	i.v.	84%	Single-choice response
14	Surgical approach	Deltopectoral	91%	Single-choice response
15	Releases	Pectoralis major muscle	47%	Multiple-choice response
16	Top 5 basic instruments	Knive		Multiple-choice response
		Electrocautery		
		Saw		
		Drill		
		Rongeur		
17	Top 5 retractors	Glenoid retractor		Multiple-choice response
		Hohmann		
		Fukuda		
		Langenbeck		
		Gelpi		
18	Cephalic vein	Retract and protect	94%	Single-choice response
19	Long head of biceps tendon	Tenodesis (soft-tissue)	77%	Single-choice response
20	Handeling the subscapularis	Tenotomy vs. peel-off	43% vs. 38%	Single-choice response
21	Glenoid pin placement	Standard guide without planning	43%	Single-choice response
22	Subscapularis reattachment in anatomic	Transosseus with non-absorbable suture	65%	Single-choice response
23	Subscapularis reattachment in reverse	Transosseus with non-absorbable suture	58%	Single-choice response
24	Antibiotics before skin closure	No	88%	Single-choice response
25	Skin closure	Subcutaneous absorbable	38%	Multiple-choice response
26	Sling - anatomic	Yes, regular	66%	Single-choice response
27	Sling - anatomic - time	4 vs. 6 wks	34% vs. 29%	Single-choice response
28	Sling - reverse	Yes, regular	64%	Single-choice response
29	Sling - reverse - time	4 vs. 6 wks	32% vs. 20%	Single-choice response
30	ROM restriction?	Yes, ER vs. ER & AE	33% vs. 24%	Multiple-choice response
31	Post-operative pain management	NSAIDs and other non-opioids vs. interscalene nerve block	28% vs. 25%	Multiple-choice response
32	Same-day discharge management	No, discharge next morning vs. No, discharge after 2 nights in hospital	32% vs. 28%	Single-choice response
33	If not, what are the reasons?	Culture vs. anesthesia support vs. physio support	19% vs. 15% vs. 15%	Multiple-choice response
34	Prescription for ROM improvement	Physiotherapy	71%	Multiple-choice response
35	Is the rehabilitation protocol the same?	Yes, same vs. No, different.	47% vs. 53%	Single-choice response
36	X-rays post-operatively?	Yes, prior to discharge vs. Yes, immediate.	46% vs. 40%	Single-choice response
37	Follow-up X-rays?	At 6 wks of follow-up vs. at yearly follow-ups	26% vs. 22%	Multiple-choice response
38	Follow-up time?	Continuously on a yearly basis	43%	Single-choice response
39	Re-evaluation of patients beyond discharge of care	Yes	87%	Single-choice response
40	Comments			

*CT*, computed tomography; *MRI*, magnetic resonance imaging; *i.v*., intravenous; *ROM*, range of motion; *NSAIDs*, non-steroidal anti-inflammatory drugs; *ER*, external rotation; *AE*, active elevation.

Looking forward, technological advances may drive future standardization, potentially embracing routine helmet use, dual-modality imaging, and integration of virtual-reality surgical planning.^15^ Such innovations, if widely adopted, could further align perioperative practices and, most importantly, enhance patient-reported outcomes.

## Conclusion

Most shoulder surgeons worldwide adhere to a shared set of intraoperative standards, including patient positioning, surgical approach, antibiotic prophylaxis, and retractor use. However, significant variation persists in pre-operative planning software utilization, post-operative immobilization strategies, and discharge practices.

These findings indicate that while intraoperative protocols are largely standardized—forming a current global practice pattern—true consensus on pre-operative and post-operative measures remains elusive. Continued international dialog and further structured, iterative research methods, such as the Delphi consensus technique, will be essential to harmonize these aspects and guide future consensus-building initiatives for elective shoulder arthroplasty.

## Disclaimers:

Funding: No funding was disclosed by the authors.

Conflicts of interest: Farhad Moola reports that he has received consulting fees from Arthex, DePuy, and Stryker.

Roger van Riet reports that he has received consulting fees and honoraria for lectures/presentations from Acumed company; is the designer for Acumed and Jake Design; and is in the current leadership role as the SECEC president.

Any additional authors, their immediate families, and any research foundations with which they are affiliated have not received any financial payments or other benefits from any commercial entity related to the subject of this article.

## Availability of data and materials

All data have been presented in this manuscript. The data are anonymous. Further data are not available.
