# Supplementary material for: A worldwide cross-sectional survey seeks the current global practice patterns on perioperative management in elective shoulder replacement surgery
Source: JSES Int. 2026 Jul 6;10(5):101758. doi: 10.1016/j.jseint.2026.101758 (PMC13470094; doi:10.1016/j.jseint.2026.101758)
Supplement: Supplementary file 1 [file mmc1.docx]

Supplemental data: Survey questions

Elective Shoulder Replacement Surgery

Start of Block: Default Question Block

Q1 **Title: Elective Shoulder Replacement Surgery – perioperative routines and management across countries**    
Thank you for accessing the survey titled, “Elective Shoulder Replacement Surgery – perioperative routines and management across various countries”, conducted by researchers and societies from various countries e.g.:

 - XXX
 - XXX
 - XXX

 This survey is being conducted to evaluated the use of surgical instruments, patient position and other key surgical practice routines / management among orthopaedic surgeons performing elective shoulder replacement surgery. Our study will serve to update and expand upon these findings with a more comprehensive survey with respect to surgical procedural preferences.

 **Any active orthopaedic surgeon/member** of the e.g. XXX  **who has treated patients (at least one) with elective shoulder replacement within the last year is eligible to participate in the study.**

 Your feedback will be collected anonymously through an online survey and should take less than 10-15 minutes to complete.

 Participation: Your participation in this online survey is completely voluntary. You are not required to provide any personal information such as name, address, phone number or email, and you don’t have to answer any question you don’t want to. The online survey system will not record your email address or IP (internet protocol) address.

 Benefits: You will receive no benefits from participating in this research study. However, we believe the results will be beneficial to all the members from the above-mentioned associations and societies to provide context in which we practice.

 Risks: The risks of participating are low. There are no foreseeable risks involved in participating in this study other than encountered in day-to-day life.

 Confidentiality: Qualtrics does not collect identifying information such as name, email address or IP address. Therefore, your responses will remain completely anonymous. No one will be able to identify you or your answers, and no one will know whether you participated in this study.

 **By continuing the questionnaire, you are consenting to participate in the online survey and its research.**

- Agree and continue (1)
- Disagree and stop (2)

Skip To: End of Survey If Title: Elective Shoulder Replacement Surgery – perioperative routines and management across count... = Disagree and stop

| Page Break |  |
| --- | --- |

Q2 1.	How old are you? (Select one)

- <30 years (1)
- 30-40 years (2)
- >40-50 years (3)
- >50-60 years (4)
- >60-70 years (5)
- >70 years (6)

Q3 2.	How would you describe your gender?

- Male (1)
- Female (2)
- Transgender (3)
- Other (non-binary, gender-fluid, agender, …) (4) __________________________________________________
- Prefer not to say (5)

Q4 3.	Since how many years are you a fully qualified orthopaedic surgeon? (Select one)

▼ 1 (1) ... 55 (55)

Q51 4.a. In what country do you work?

▼ Algeria (55) ... Zimbabwe (122)

Q5 4.b. What type of work setting do you work in? (Select all that apply)

- University hospital (1)
- General hospital (2)
- Private hospital (3)
- Ambulatory surgery center (4)
- Private practice (5)
- Other (6) __________________________________________________

Q6 5.	How many years of work experience do you have? (Select one)

- < 5 (1)
- 5 – 10 (2)
- 10 – 20 (3)
- 20 – 30 (4)
- > 30 (5)

Q7 6.	Do you have fellowship/extended subspecialty training in shoulder surgery (e.g. shoulder and elbow, upperlimb surgery,…)? (Select one)

- Yes (1)
- No (2)

Q8 7.	How many shoulder arthroplasties (anatomic + reverse) do you do per year?

- <20 (1)
- 20-50 (2)
- 50-100 (3)
- 100-200 (4)
- 200-300 (5)
- >300 (6)

End of Block: Default Question Block

Start of Block: Block 1

Q9 8.	What additional routine preoperative imaging to x-rays do you obtain for an elective shoulder replacement? (Select all that apply)

- Ultrasound (1)
- CT scan (2)
- MRI scan (3)
- Other (4) __________________________________________________

Q10 9.	How do you routinely ascertain rotator cuff pathology in respect of performing an anatomic or reverse shoulder replacement? (Select one)

- Physical examination (1)
- Ultrasound (2)
- X-ray (3)
- CT scan (4)
- MRI scan (5)
- Intraoperative decision (6)
- Other (7) __________________________________________________

Q11 10.	Do you use a CT planning software prior surgery? (Select one)

- No (1)
- Yes, routinely (2)
- Yes, difficult glenoids (3)
- Yes, revision cases (4)

| Page Break |  |
| --- | --- |

Q12 11.	How do you usually position the patient for shoulder arthroplasty? (Select one)

- Supine (flat) (1)
- Lazy beach chair (approx. 20° incline) (2)
- Beach chair (3)
- Lateral (4)
- Prone (5)
- Other (6) __________________________________________________

Q13 12.	What type of arm holding device do you routinely use during the surgical procedure? (Select one)

- None (1)
- Pneumatic arm holder (e.g. Spider, Trimano…) (2)
- Arm rest (3)
- Mayo stand (4)
- Assistant (5)
- Other (6) __________________________________________________

Q14 13.	Do you routinely use a skin cleanser prior surgery? (Select one)

- No (1)
- Yes, soap (2)
- Yes, Benzyl peroxide (3)
- Yes, benzyl peroxide with clindamycin (4)
- Yes, chlorhexidine (5)
- Yes, other (6) __________________________________________________

Q15 14.	After cautious standard prep and draping procedures, do you use additional precautions to minimize infection risk? (Select all that apply)

- No (1)
- Integuseal (Microbial sealant) (2)
- Ioban drape (3)
- Helmet use during procedure (4)
- Hydrogen peroxide (5)
- Other (6)

| Page Break |  |
| --- | --- |

Q16 15.	Do you routinely use perioperative antibiotics? (Select one)

- No (1)
- Yes, single shot preoperatively (2)
- Yes, preoperatively up to post x24 h (3)
- Yes, preoperatively up to post op x48h (4)
- Yes, preoperatively until wound healing (5)
- Yes, preoperatively until etc (6)
- Yes, other (7) __________________________________________________

Display This Question:

If 15. Do you routinely use perioperative antibiotics? (Select one) != No

Q17 15.1. If yes, what antibiotics to routinely use? (Select one)

- Cefazolin (1)
- Vancomycin (2)
- Clindamycin (3)
- Other (4) __________________________________________________

Q18 16.	Do you use tranexamic acid (TXA)? (Select one)

- No (1)
- Yes, routine (2)
- Yes, occasional (3)

Display This Question:

If 16. Do you use tranexamic acid (TXA)? (Select one) != No

Q19 16.1 If yes, when is tranexamic acid (TXA) administered? (Select one)

- Start (1)
- Midway (2)
- End of operation (3)

Display This Question:

If 16. Do you use tranexamic acid (TXA)? (Select one) != No

Q20 16.2 If yes, how is TXA administered? (Select one)

- Topical (1)
- IV (2)
- IV and topical (3)

End of Block: Block 1

Start of Block: Block 2

Q21 17.	What is your standard surgical approach? (Select one)

- Delta-split (1)
- Anterolateral (2)
- Deltopectoral (3)
- Posterior (4)
- Other (5) __________________________________________________

Q22 18.	Do you release any of the following structures by a few millimeters routinely to improve exposure? (Select all that apply)

- Pectoralis major muscle (1)
- Coracoacromial ligament (2)
- Latissimus dorsi muscle (3)
- Other (4) __________________________________________________
- No (5)

Q23 19.	What are the basic instruments (non-retractors) on your tray that you routinely use during your procedure? (Select all that apply)

- Knive with various blades (#10, 15, 20, 21, ...) (1)
- Adson forceps (2)
- Bonney forceps (3)
- Gillies forceps (4)
- Debakey forceps (5)
- Mayo scissors (6)
- Metzenbaum scissors (7)
- Electrocautery (8)
- Cobb elevator (9)
- Freer elevator (10)
- Raspatory (blunt & sharp) (11)
- Rongeur (Bone Leur) (12)
- Osteotomes (13)
- Chisels (14)
- Saw (15)
- Drill (16)
- Bone hook (17)
- Other (18) __________________________________________________

| Page Break |  |
| --- | --- |

Q24 20.	What type of “retractors” do you routinely use during surgical exposure? (Select all that apply)

- Alexis wound sleeve (1)
- Army-Navy (2)
- Bell-Hawkins Shoulder Frame (3)
- Brown (4)
- Churchhill (5)
- Darrach (straight/bent, narrow + wide) (6)
- Fukuda (7)
- Glenoid retractors (spiked narrow + wide) (8)
- Gelpi (Subsscapularis spreader) (9)
- Hohmann (blunt & spiked) (10)
- Kelly (11)
- Kilner skin retractor (cat paw) (12)
- Kobel (13)
- Kocher (14)
- Langenbeck (15)
- Richardson (16)
- Roux (17)
- Sonnabend (18)
- Volkmann (cat claw) (19)
- Weitlaner wound spreader (20)

Q25 21. If you had to select **your 5 most important “retractors”** for your routine surgical exposure of an elective shoulder arthroplasty, which would it be? (Select 5)

- Alexis wound sleeve (1)
- Army-Navy (2)
- Bell-Hawkins Shoulder Frame (3)
- Brown (4)
- Churchhill (5)
- Darrach (straight/bent, narrow + wide) (6)
- Fukuda (7)
- Glenoid retractors (spiked narrow + wide) (8)
- Gelpi (Subsscapularis spreader) (9)
- Hohmann (blunt & spiked) (10)
- Kelly (11)
- Kilner skin retractor (cat paw) (12)
- Kobel (13)
- Kocher (14)
- Langenbeck (15)
- Richardson (16)
- Roux (17)
- Sonnabend (18)
- Volkmann (cat claw) (19)
- Weitlaner wound spreader (20)
- Other (21) __________________________________________________

| Page Break |  |
| --- | --- |

Q26 22.	What do you routinely do with the cephalic vein during approach? (Select one)

- Retract and protect (1)
- Ligate (2)
- Other (3) __________________________________________________

Q27 23.	What do you routinely do with the long head of biceps tendon? (Select one)

- Tenotomy (1)
- Tenodesis – soft tissue (2)
- Tenodesis – anchor/screw (3)
- Leave attached (4)
- Other (5) __________________________________________________

Q28 24.	How do you routinely treat the subscapularis during the approach? (Select one)

- Tenotomy (1)
- Peel-off (2)
- Lesser tubercle osteotomy (3)
- Subscapularis-split (4)
- Nothing, I use a posterior approach (5)
- Nothing, I use a subscapularis sparing approach (7)
- Other (8) __________________________________________________

Q29 25.	After glenoid exposure, what do you routinely use for central pin placement for glenoid component preparation? (Select one)

- Freehanded pin placement **without** prior use of pre-operative (3d) planning software (1)
- Freehanded pin placement **with** prior use of pre-operative (3d) planning software (2)
- Standard guide for pin placement from the implant company **without** prior use of pre-operative (3d) planning software (3)
- Standard guide for pin placement from the implant company **with** prior use of pre-operative (3d) planning software (7)
- Patient specific instrument (PSI) – guide/jig, 3d print and sterilized (4)
- Real time surgical guidance/navigation (5)
- Other (6) __________________________________________________

| Page Break |  |
| --- | --- |

Q30 26. How do you routinely treat/reattach the subscapularis tendon during closure of **an anatomic shoulder replacement**? (Select one)

- Tendon end-to-end/side-to-side refixation (1)
- Transosseous refixation with non-absorbable sutures (2)
- Transosseous refixation with absorbable sutures (3)
- Cerclage wire (4)
- No refixation (5)
- Other (6) __________________________________________________

Q31 27. How do you routinely treat/reattach the subscapularis tendon during closure of **a reverse shoulder replacement**? (Select one)

- Tendon end-to-end/side-to-side refixation (1)
- Transosseous refixation with non-absorbable sutures (2)
- Transosseous refixation with absorbable sutures (3)
- Cerclage wire (4)
- No refixation (5)
- Other (6) __________________________________________________

Q32 28. Do you apply local/topical antibiotics during closure? (Select one)

- No (1)
- Yes, vancomycin powder (2)
- Yes, vancomycin injection (3)
- Yes, other (4) __________________________________________________

Q33 29.	How do you routinely perform final skin closure before putting on a dressing? (Select all that apply)

- Subcuticular – absorbable suture (1)
- Subcuticular – non-absorbable suture (2)
- Extracuticular – absorbable suture (3)
- Extracuticular – non-absorbable suture (4)
- Staples (5)
- Glue (6)
- Steri-strips (7)
- Other (8)

| Page Break |  |
| --- | --- |

Q34 30. Do you routinely use a sling after **an anatomic shoulder replacement** and advice patients to use it postoperatively? (Select one)

- Yes, regular sling. (1)
- Yes, abduction sling (2)
- No. (4)
- Other (5) __________________________________________________

Display This Question:

If 30. Do you routinely use a sling after an anatomic shoulder replacement and advice patients to us... = Yes, regular sling.

Or 30. Do you routinely use a sling after an anatomic shoulder replacement and advice patients to us... = Yes, abduction sling

Q35 30.a. If you use a sling after an anatomic replacement, how long do you use it for (weeks)?

▼ 1 (1) ... 20 (20)

Display This Question:

If 30. Do you routinely use a sling after an anatomic shoulder replacement and advice patients to us... = No.

Q41 30.b. If you do not use a sling with an anatomic replacement postoperatively, why? What do you do?

________________________________________________________________

Q37 31. Do you routinely use a sling after **a reverse shoulder replacement** and advice patients to use it postoperatively? (Select one)

- Yes, regular sling. (1)
- Yes, abduction sling. (2)
- No. (4)
- Other (5) __________________________________________________

Display This Question:

If 31. Do you routinely use a sling after a reverse shoulder replacement and advice patients to use... = Yes, regular sling.

Or 31. Do you routinely use a sling after a reverse shoulder replacement and advice patients to use... = Yes, abduction sling.

Q40 31.a. If you use a sling after a reverse replacement, how long do you use it for (weeks)?

▼ 1 (1) ... 20 (20)

Display This Question:

If 31. Do you routinely use a sling after a reverse shoulder replacement and advice patients to use... = No.

Q36 31.b. If you do not use a sling with a reverse replacement postoperatively, why? What do you do?

________________________________________________________________

Q42 32.	Do you restrict ROM for patients within the first 6 weeks? (Select all that apply)

- No (1)
- Yes, no external rotation (ER) only (2)
- Yes, no ER and active elevation (3)
- Yes, only pendulum exercises allowed (4)
- Yes, … (5) __________________________________________________
- Other… (6) __________________________________________________

| Page Break |  |
| --- | --- |

Q43 33.	How do you routinely manage immediate postoperative pain within first 5 days? (Select all that apply)

- Patients receive an interscalene nerve block (1)
- LIA – Local Infiltration Anesthetic (2)
- Opiods for 2-3 days (3)
- Opiods for as long as it takes (4)
- NSAIDs and other non-opiods (5)
- Local cooling device (6)
- Other (7) __________________________________________________

Q44 34.	Do you routinely discharge patients with an anatomic or reverse shoulder replacement on day of surgery – same day surgery (SDS)? If not, how many days do you keep them hospitalized until discharge? (Select one)

- Yes. (1)
- No, discharge next morning. (2)
- No, discharge after 2 nights in hospital. (3)
- No, after 3-5 nights. (4)
- No, more than 5 nights (5-10 days). (5)
- No, when wound has healed (>10 days). (6)
- Other (7) __________________________________________________

Display This Question:

If 34. Do you routinely discharge patients with an anatomic or reverse shoulder replacement on day o... != Yes.

Q45 34.a If you do not discharge the patient the same day of surgery, why? What is preventing you to change it?

- Hospital resources (1)
- Culture (2)
- Billing system (lower reimbursement) (3)
- Anesthesia support (4)
- Physio support (5)
- Patient travel distances (6)
- Other logistics (7)
- Other (8) __________________________________________________

Q46 35. What do you prescribe postoperatively to improve range of motion (ROM)? (Select all that apply)

- Nothing (1)
- Yes, physiotherapy (2)
- Yes, continuous passive motion devices (3)
- Yes, aqua- exercise after wound healing (4)
- Other (5) __________________________________________________

Q47 36.	Is your postop rehabilitation same following reverse versus anatomic total shoulder replacement? (Select one)

- Yes, same. (1)
- No, different. (2)

| Page Break |  |
| --- | --- |

Q48 37.	Do you routinely obtain x-rays during hospital admission/same day surgery? (Select one)

- No (1)
- No, only when there is problem (2)
- Yes, immediate (3)
- Yes, prior to discharge (4)
- Other… (5) __________________________________________________

Q49 38.	When do you routinely obtain x-rays during follow-up? (select all that apply)

- At 2 weeks follow-up (1)
- At 6 weeks follow-up (2)
- At 12 weeks follow-up (3)
- At 6 months follow-up (4)
- At yearly follow-ups (5)
- I only obtain x-rays in the follow-up, if there is an issue (6)
- Other (7) __________________________________________________

Q50 39.	How long do you follow up your patients post shoulder replacement? (Select one)

- Until post-operative discharge from hospital/surgery center (1)
- Until wound healing (2)
- Up to 6 weeks (3)
- Up to 3 months (4)
- Up to the first year (5)
- Up to two years (6)
- I monitor them continuously on a yearly basis (7)
- Other (8) __________________________________________________

Q51 40.	Will you see back your all shoulder replacement patients for reevaluation beyond discharge of care? (Select one)

- Yes. (1)
- No. (2)
- I redirect their referral to a revision surgeon. (3)
- Other. (4)

Q52 Further comments – Thoughts/ideas that you like to share with us?

________________________________________________________________

End of Block: Block 2
